# Vaginal Hysterectomy of the Didelphic Uterus

**DOI:** 10.7759/cureus.64767

**Published:** 2024-07-17

**Authors:** Madeline White, Howard Herrell

**Affiliations:** 1 Obstetrics and Gynecology, Ballad Health, Greeneville, USA; 2 Obstetrics and Gynecology, Greeneville Community Hospital, Greeneville, USA

**Keywords:** mullerian abnormalities, morcellation, total vaginal hysterectomy, vaginal hysterectomy, didelphic uterus

## Abstract

Hysterectomy of the didelphic uterus is extensively documented utilizing the laparoscopic and abdominal surgical approach with very few cases documenting the vaginal approach. This report will discuss the surgical modifications to the vaginal hysterectomy (VH) technique successfully employed in a case of a didelphic uterus. These techniques include staged transection of the uterosacral ligaments for the difficult posterior colpotomy, morcellation for the enlarged size of the uterus, and inversion of the uterus for delivery through the vaginal canal. This case displays the feasibility of the vaginal route for the didelphic uterus and augments the American College of Obstetricians and Gynecologists (ACOG) Committee Opinion No. 701 by expanding the patient pathology that qualifies for benign indications for hysterectomy through the most minimally invasive route.

## Introduction

A didelphic uterus results from the failure of the Müllerian ducts to fuse together during embryological development. This can result in a complete double uterus, double cervix, and double vagina with a longitudinal septum. When embryological structures fail to develop properly, it is important to look for coexisting anomalies because they can pose potential challenges with hysterectomy.

A Finnish retrospective study quantified renal variants in females with didelphic uterus and found that 29.1% had coexisting renal tract malformation [1]. The most common renal anomaly documented in those females was unilateral renal agenesis. A rare but important variant is the Herlyn-Werner-Wunderlich syndrome (HWWS), which is characterized by a didelphic uterus, obstructed hemivagina, and ipsilateral renal agenesis [2]. Other potential anatomical variants exist, and preoperative categorization is important. Preoperative knowledge of the location of the bladder and ureter and their proximity to the uterus is key to performing safe surgery for these variants.

A review of ultrasound imaging of didelphic uteri cases has shown the bladder to be in a normal anatomical location, anterior and inferior to the combined horns of each uterus [3]. The Finnish study found no association between didelphic uteri and duplex system ureteric variants such as bifid or double ureters [1].

Surgeons should have a high suspicion of coexisting anatomical variants prior to planning surgery.

For patients requiring hysterectomy for benign indications, vaginal hysterectomy (VH) is the procedure of choice unless there are specific contraindications. When comparing potential routes of hysterectomy for benign indications, VH is the least invasive option, produces the fewest complications, has the lowest direct and indirect cost to the patient and provider, and has the fastest recovery time for the patient [4]. The American College of Obstetricians and Gynecologists recommends VH as the preferred choice for benign cases [5].

In this report, we discuss a case of VH utilized for a patient with a didelphic uterus to support its role as a safe and acceptable route of hysterectomy as many surgeons might be concerned about the applicability of the technique for this pathology.

## Case presentation

The patient was a gravida 4 para 2 female in her late 40s with a diagnosis of didelphic uterus who presented with a history of irregular menstrual patterns and dysmenorrhea worsening over the past three months. Conservative treatments with oral progesterone and depot medroxyprogesterone acetate were previously attempted without any improvement in her condition. Her surgical history included a vaginal septum resection as an elective procedure and two cesarean deliveries. An endometrial biopsy and transvaginal ultrasound were ordered to determine the size of the didelphic uterus and the size of the existing fibroids and to exclude malignancy of the endometrium. The transvaginal ultrasound performed prior to the hysterectomy showed the whole uterus measuring 9.8×9×9.4 cm with a volume of 437 mL. The right uterus measured 9.7×9.1×7.2 cm and contained a large lateral fibroid measuring 7.2×6.5×6.5 cm. The left uterus measured 9×4.5×4.3 cm and contained a small fundal fibroid measuring less than 2 cm. Speculum examination revealed two cervices. A transabdominal ultrasound performed years prior, at a separate institution, showed the kidneys to have normal size and echotexture with no indications of abnormality. Chart review showed a prior ultrasound of the renal system with normal renal anatomy.

Considerations going into surgery

The patient had two prior cesareans and a large fibroid that could contribute to a lack of uterine descent and require morcellation. Normal VH techniques for posterior and anterior colpotomy rely on a well-defined cervix and lower uterine segment that is absent in this case.

Surgical procedure

Initial Approach

The cervices were held en masse and treated as one with the tenaculum. Vasopressin was injected into the cervix circumferentially. A circumferential incision around both cervices was made, and the anterior vaginal wall mucosa was pushed cephalad.

Difficult Posterior Colpotomy

The posterior incision proved difficult as the posterior cul-de-sac peritoneal reflection line was not easily visualized. The lack of descent of the uterus allowing visualization of the peritoneal reflection can be attributed to the larger size of the uterus. To gain greater descent of the uterus, a staged transection of the uterosacral ligaments was performed. An energy sealing device was used to take the upper portions of the uterosacral ligaments first to gain uterine descent. The peritoneal reflection was then identified, and the posterior cul-de-sac was sharply entered with Mayo scissors at a 90-degree angle to the cervix, creating the posterior colpotomy. The posterior peritoneum was tagged to the posterior vaginal wall. With partial descent of the uterus, the uterosacral ligaments were clamped, transected, and suture ligated completely. When securing and dividing the uterosacral ligaments in this poorly descended uterus, the clamp can come as close as 1 cm to the ureter [4]. Placing the clamp close to the cervix and avoiding the lateral roll of the tip was utilized to avoid injury to the ureter.

Morcellation for Mass Reduction

Due to the size of the uterus, morcellation was performed by serial wedge resections to debulk the uterus. This occurred after the uterine arteries had already been taken with the energy sealing device. The tenaculum holding the cervices as one unit was removed, and a tenaculum was placed on each individual cervix. Using a scalpel, the two cervices were separated, and wedge resection was performed on the right hemiuterus. The 7 cm fibroid on the right was identified, debulked, and removed posteriorly. The smaller fibroid was identified and removed from the left uterus. Morcellation debulked the uterus sufficiently and allowed adequate access to the upper pedicles for sealing with the energy sealing device and easy delivery through the vaginal vault. Visualization before every cut was performed to avoid cutting through the wall of the uterus with the scalpel. The serosa of the uterus was kept intact to avoid possible injury to other viscera. Once the uterus was adequately debulked, the normal hysterectomy technique resumed.

Inversion Technique

The anterior colpotomy was made without encountering scar tissue related to prior cesarean deliveries. The rest of the procedure comprised normal techniques without the need for modifications. The removal of the uterus through the vaginal canal utilized the inversion technique. The posterior wall of the uterus was grasped with a tenaculum, and the cervix was pushed through the anterior colpotomy, resulting in the fundus being delivered through the posterior colpotomy where it could be grasped with a new tenaculum. The upper pedicles were then sealed and divided with the energy sealing device. This protects the abdominal cavity and peritoneal structures from thermal injury and allows easier access to the fallopian tubes for removal.

Bilateral salpingectomy followed by a modified McCall culdoplasty and cuff closure was performed to complete the procedure.

Outcome and follow-up

**Figure 1:**
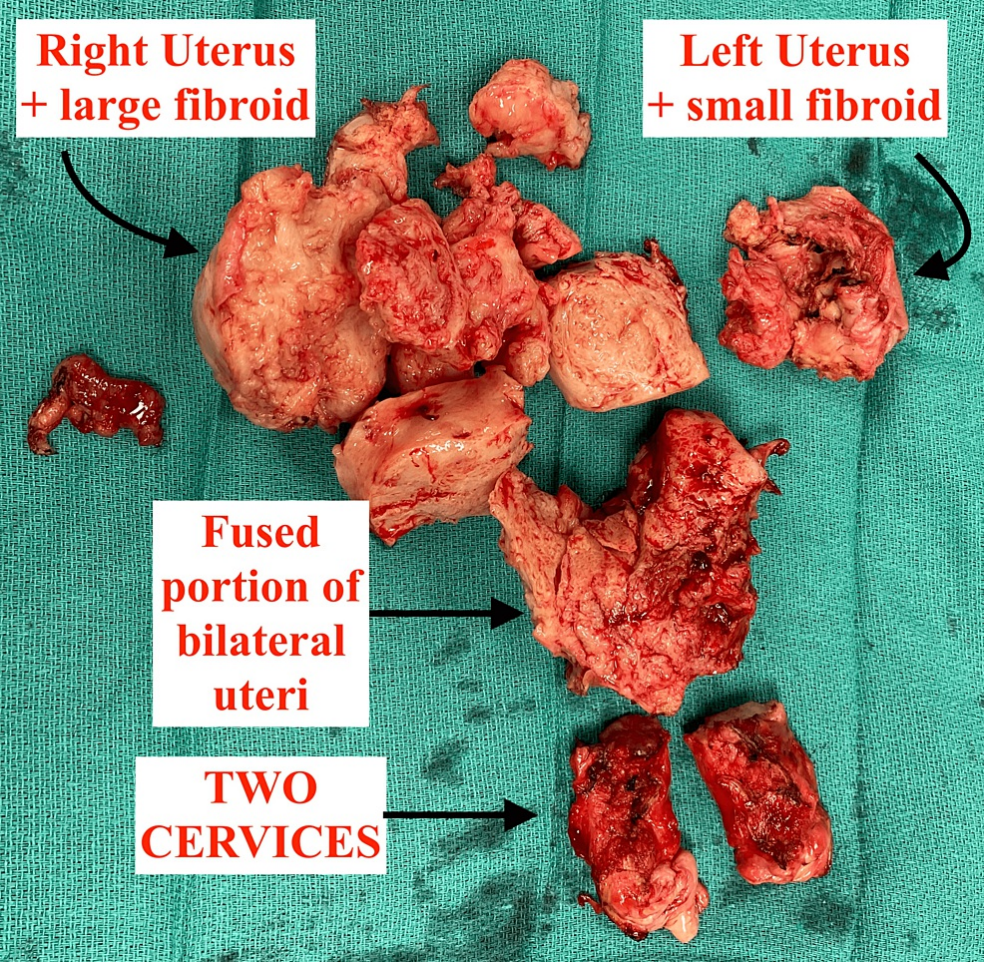
Morcellated uterine specimen weighing 408 g with evidence of adenomyosis and multiple leiomyomata

The patient underwent a total VH with an operating time of 58 minutes. Pathology revealed a 408 g uterine specimen with evidence of adenomyosis and multiple leiomyomata (fibroids) as seen in Figure 1.

The procedure was done on an outpatient basis, and she was discharged home approximately four hours after the completion of surgery. She returned to normal activity within two days and was followed up in our outpatient office at two and six weeks postoperatively with no concerns.

## Discussion

It is important to recognize when a successful minimally invasive procedure can be done in unique cases. Vaginal hysterectomy is a safe option for didelphic uteri with minimal modifications. VH is the optimal choice in terms of cost, recovery time, and complication rates [4,6]. The notable FINHYST study was a prospective cohort study that looked at 5,279 benign hysterectomies and the complications and risk factors associated with each type: abdominal hysterectomy (AH), vaginal hysterectomy (VH), and laparoscopic hysterectomy (LH). Patients undergoing VH in comparison to LH and AH experienced lower rates of major complications at 2.6%, 4.3%, and 4%, respectively. The FINHYST study found that in cases for benign indications, VH carried the lowest risk of bladder, ureter, and bowel injury in comparison to other routes [6].

Utilizing techniques that minimize the difficult hysterectomy due to an enlarged uterus, a uterus that does not descend, or a didelphic uterus can maximize beneficial outcomes for the patient and the surgeon. Some of the techniques discussed require repetition and have a learning curve, but they can be taught and utilized to make VH possible in the case of a didelphic or enlarged uterus.

We found one previous report from 2018 of a successful VH for a didelphic uterus [7]. A literature review revealed that other case reports of hysterectomy for the didelphic uterus are predominantly by the endoscopic or laparotomy route [8-10]. The patient outcome in this report reinforces the 2018 finding that VH is a possibility for patients with a didelphic uterus [7]. We believe that experienced vaginal surgeons have probably performed this before, but the documentation of such cases is sparse throughout the literature.

It should be stated that while this case was started and completed through the vaginal route, there is a possibility that some cases might have unforeseen obstacles that require conversion to another approach. Surgeons should be comfortable with the possibility of conversion when attempting more difficult pathology by the vaginal route. If progress cannot be made, a transition to a laparoscopic approach is an acceptable and well-established option. Complex anatomy should not deter the surgeon from offering the patient the least invasive option. The surgical techniques discussed in this case make the approach relatively straightforward. Future research should focus on a comparison of outcomes from different routes of hysterectomy for didelphic uteri.

It is worth mentioning that the surgeon who performed this procedure is a skilled vaginal surgeon. The art of vaginal hysterectomy is often described as a "dying art," which we, the authors, feel is a crisis in gynecology as VH is the preferred method of hysterectomy for benign cases. This case has been described by distant colleagues as "lucky" for not having any bad outcomes and "brave" for being attempted in the first place. We disagree with both of these statements. Patients should always be offered the opportunity to have the most minimally invasive procedure for their problem, and unusual anatomy should not be feared by the surgeon; rather, it should inspire them to make modifications such as those mentioned in this paper to complete the surgery in a safe and successful manner [5]*.*

## Conclusions

Müllerian abnormalities are historically associated with coexisting anomalies. It is important to keep those in mind and review patient history and imaging prior to beginning surgery. Vaginal hysterectomy for the didelphic uterus can be successfully completed with modifications to surgical technique including staged transection of the uterosacral ligaments, morcellation for mass reduction, and inversion of the uterus for delivery through the vaginal canal. The indications for each method of hysterectomy we perform on patients should constantly be expanding with the most minimally invasive option remaining a priority.
